# An improved chloride-conducting channelrhodopsin for light-induced inhibition of neuronal activity in vivo

**DOI:** 10.1038/srep14807

**Published:** 2015-10-07

**Authors:** Jonas Wietek, Riccardo Beltramo, Massimo Scanziani, Peter Hegemann, Thomas G. Oertner, J. Simon Wiegert

**Affiliations:** 1 Institute for Biology, Experimental Biophysics, Humboldt-Universität zu Berlin, D-10115 Berlin, Germany; 2 Center for Neural Circuits and Behavior, University of California San Diego, La Jolla, CA 92093-0634, USA; 3 Howard Hughes Medical Institute, University of California San Diego, La Jolla, CA 92093-0634, USA; 4 Institute for Synaptic Physiology, Center for Molecular Neurobiology Hamburg, University Medical Center Hamburg-Eppendorf, D-20251 Hamburg, Germany

## Abstract

Channelrhodopsins are light-gated cation channels that have been widely used for optogenetic stimulation of electrically excitable cells. Replacement of a glutamic acid in the central gate with a positively charged amino acid residue reverses the ion selectivity and produces chloride-conducting ChRs (ChloCs). Expressed in neurons, published ChloCs produced a strong shunting effect but also a small, yet significant depolarization from the resting potential. Depending on the state of the neuron, the net result of illumination might therefore be inhibitory or excitatory with respect to action potential generation. Here we report two additional amino acid substitutions that significantly shift the reversal potential of improved ChloC (iChloC) to the reversal potential of endogenous GABA_A_ receptors. As a result, light-evoked membrane depolarization was strongly reduced and spike initiation after current injection or synaptic stimulation was reliably inhibited in iChloC-transfected neurons *in vitro*. In the primary visual cortex of anesthetized mice, activation of iChloC suppressed spiking activity evoked by visual stimulation. Due to its high operational light sensitivity, iChloC makes it possible to inhibit neurons in a large volume of brain tissue from a small, point-like light source.

Optogenetic stimulation of neurons with channelrhodopsin variants has been applied to many neurobiological questions in a wide variety of organisms and model systems. It has been especially useful to dissect and manipulate the brain circuitry of small rodents and to derive neuronal correlates of behavioral adaptations and disorders^1^,^2^,^3^,^4^. While optogenetic activation is widely used, better tools for optogenetic inhibition of neuronal activity are wanted, as loss-of-function experiments are often more specific and easier to interpret in the context of a complex system like the brain. Inhibition of neuronal activity, however, has proven to be technically difficult. So far, the only inhibitory tools that were successfully applied *in vivo* are the light-driven chloride pump Halorhodopsin^5^ and the proton pump Archaerhodopsin^6^. Both pumps require dense expression in the plasma membrane and high light intensities for reliable inhibition of neuronal activity as only a single ion is transported per absorbed photon. During prolonged activation, pumps affect the intracellular ion composition and can severely change the effects of endogenous GABAergic inhibition^7^. Recently engineered light-gated chloride channels^8^,^9^ (here commonly referred to as ChloCs) are in principle much more efficient than pumps since thousands of ions can pass the channel per absorbed photon. The operational light sensitivity is further increased by the slow off-kinetics of some ChloC variants, sustaining inhibition for several seconds. High light sensitivity is an important feature if large volumes of neuronal tissue are to be addressed from a localized light source such as a fiber-coupled laser or LED. The reversal potential (E_rev_) of published ChloCs, however, is not quite as negative as E_rev_ of endogenous chloride channels (e.g. GABA_A_ receptors), indicating a residual conductance for cations^8^. Given the dramatic developmental changes in [Cl^−^]_i_^10^ and its dependence on the local concentration of impermeant anions^11^, the net effect of published ChloC variants on neuronal excitability *in vivo* is difficult to predict.

Here, in an attempt to improve the ion selectivity of ChloC, we combine the three point mutations of slowChloC^8^ with two additional amino acid substitutions to reduce the number of negative charges protruding into the water pore. In neurons, photocurrents of our improved ChloC (iChloC) reverse at nearly identical membrane potentials than GABAergic IPSCs, suggesting a high selectivity for Cl^−^ ions. iChloC no longer shows depolarizing activity in patch-clamped neurons and reliably inhibits synaptically evoked spikes in undisturbed CA1 pyramidal cells. When expressed in primary visual cortex, iChloC strongly suppressed visually evoked spiking of pyramidal neurons *in vivo*. Thus, due to its high Cl^−^-selectivity and operational light sensitivity, iChloC is an ideal tool to silence neurons *in vivo* with very low light exposure.

## Results

**Figure 1:**
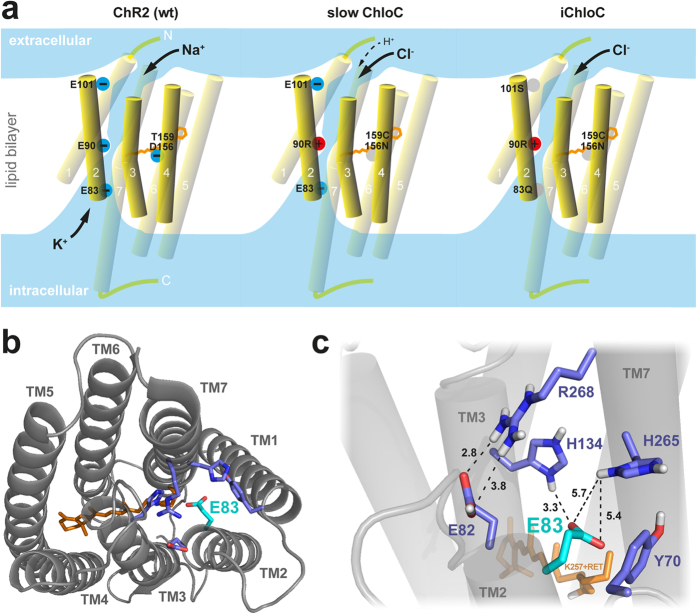
Strategy for improving the chloride selectivity of ChloC. (**A**) Position of key negative (blue) and positive charges (red) in wt Channelrhodopsin, slowChloC, and iChloC with respect to the aqueous pore (light blue)^14^. (**B)** Structural model of ChR2-E90R^8^; view from the intracellular side. Residues having a stake in the inner gate are highlighted in blue and cyan (TM: transmembrane helix). The negatively charged E83 carboxylate is located in the center of the inner gate pore. **(C)** Closer look at the inner gate residues (side view). Hydrogen bonds may form between E82 and R268 as well as between E83 and H134. Atomic distances (black dotted lines) are shown in Å. The retinal (RET) is shown in orange.

**Figure 2:**
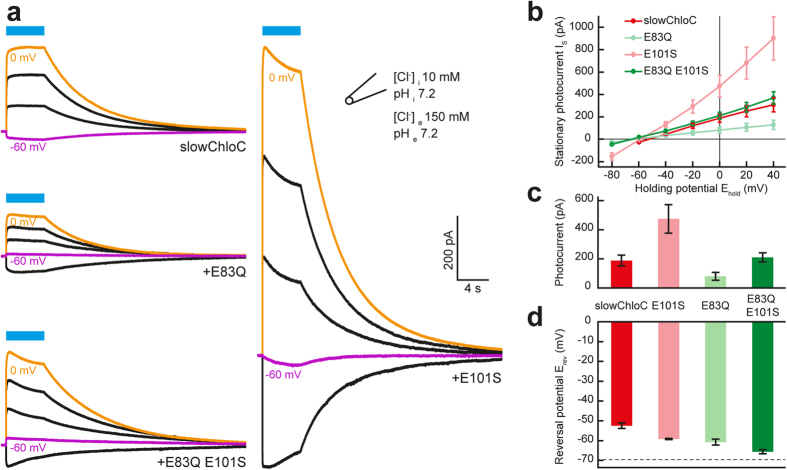
Characterization of slowChloC variants in HEK293 cells. (**A**) Photocurrent example traces of slowChloC, slowChloC (E101S), slowChloC (E83Q) and slowChloC (E83Q, E101S) (iChloC) in HEK 293 cells at different holding potentials (20 mV steps). **(B)** I-E curves for the 4 ChloC variants. **(C)** Photocurrent amplitudes for the 4 ChloC variants measured at a holding potential of 0 mV. **(D)** Reversal potential in HEK 293 cells (slowChloC, −52.5 ± 1.3 mV; slowChloC (E101S), −59.2 ± 0.3 mV; slowChloC (E83Q), −60.8 ± 1.5 mV; iChloC, −65.6 ± 1.1 mV). Dashed line indicates calculated Nernst potential for Cl^−^ (−69.6 mV). n = 9 for slowChloC, n = 8 for slowChloC (E101S), n = 6 for other two variants.

The previously published slowChloC carries a Glutamate-to-Arginine substitution at position 90 (E90R), introducing a positive charge to the central gate of the protein. In addition, Threonine 159 was mutated to Cysteine (T159C) to increase photocurrents^12^ and Aspartic acid 156 was substituted with Asparagine (D156N) to stabilize the open state of the channel and increase its operational light sensitivity^13^. To further increase the selectivity of the channel for Cl^−^ and to suppress cation conductance, we attempted to render the inner gate as well as the^9^ extracellular access channel more permissive for anions. Residue E83 is a key component of the inner gate, forming a hydrogen bond to H134 in ChR2^14^,^15^. In our ChR2-E90R model^8^, E83 extends its negatively charged carboxylate group into the pore, thus forming a potential diffusion barrier for anions (Fig. 1A & B). In addition, E83 is part of the proton transfer chain and its replacement reduces cation conductance in ChR2 [15, 16]. To eliminate the negative charge at this position without disturbing the overall geometry, we replaced E83 with a Glutamine (Q) residue (Fig. 1A, B and C). The resulting ChloC variants were characterized in HEK 293 cells. Compared to slowChloC, the E83Q replacement shifted the reversal potential by approx. −8 mV (E_rev_ slowChloC[E83Q] = −60.8 ± 1.5 mV), but reduced current amplitudes from 188 ± 38 pA to 80 ± 27 pA (Fig. 2). E101, which is positioned at the collar of the vestibule facing the extracellular space^14^, also carries a negative charge that may hinder anion diffusion. In addition, E101, like E83, is a constituent of the proton transfer chain and deletion of its side chain (E101A) reduced cation conductance^16^,^17^. When we replaced E101 in the access channel with a neutral Serine (S), we obtained very large photocurrents (475 ± 98 pA) with a reversal potential of –59.2 ± 0.3 mV, suggesting that anion conductance was increased (Fig. 2). When we combined the two mutations, we measured a current amplitude of 210 ± 32 pA and an additional shift of the reversal potential (E_rev _= −65. 6 ± 1.1 mV) to a value 13 mV more negative than slowChloC (E_rev _= −52.5 ± 1.3 mV) and close to the calculated Nernst potential for Cl^−^ (−69.6 mV, Fig. 2D). The improved chloride-conducting channelrhodopsin ChR2(E83Q,E90R,E101S,D156N,T159C) we termed iChloC.

**Figure 3:**
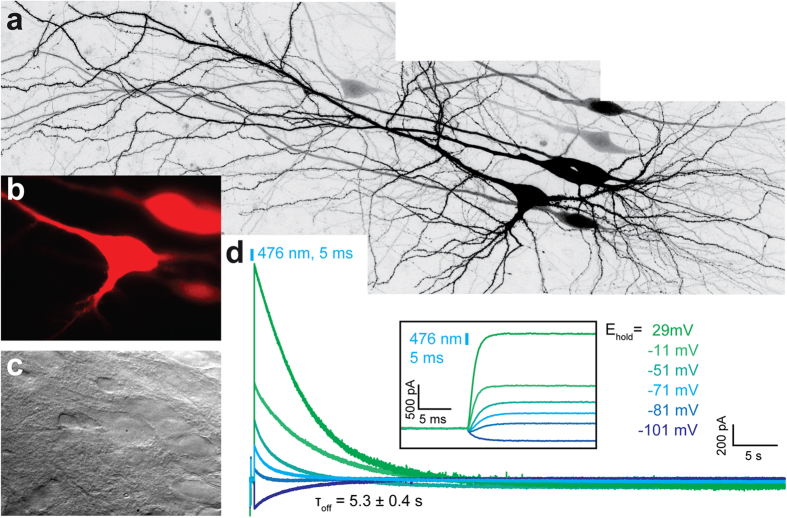
Expression of iChloC in CA1 pyramidal cells in organotypic hippocampal slice culture. (**A**) Overview of neuronal morphology 5 days after electroporation (maximum intensity projection of two-photon images). (**B)** Fluorescence of co-expressed tdimer2 was used to target transfected neurons for electrophysiological recordings (same cells as in (**A**)). **(C)** Dodt contrast image of cells shown in (**B**). **(D)** Photocurrents in response to a single light pulse (476 nm, 5 ms, 1 mW/mm^2^) at different holding potentials. Photocurrents reversed at very negative holding potentials and were large at depolarized holding potentials, where the Cl^−^ inward driving force was highest (same neuron as in (**A**–**C**)). The inset shows the onset of the photocurrents at higher temporal resolution. Indicated holding potentials were rounded to full numbers after subtraction of the liquid junction potential (−10.6 mV). Indicated tau value was derived from 9 independent measurements in 9 slice cultures.

For neuron-specific expression, we assembled an expression plasmid driven by the human CaMKII-promotor, including a red florescent protein (tdimer2) behind a ribosomal skip sequence (CaMKII -iChloC-2A-tdimer2). The cytoplasmic tdimer2 allows straightforward identification of transfected neurons and evaluation of their morphology. Four to five days after single cell electroporation, expressing CA1 pyramidal cells could be readily identified by their red fluorescence and were indistinguishable from non-transfected neurons under Dodt contrast^18^ (Fig. 3B,C). Dendritic morphology and electrophysiological properties showed no abnormalities (Fig. 3A, Table 1). Brief light pulses (476 nm, 5 ms, 1 mW/mm^2^) induced large photocurrents in transfected neurons with a decay time constant of 5.3 ± 0.4 s (Fig. 3D). Holding potentials were corrected for a liquid junction potential of −10.6 mV.

**Figure 4:**
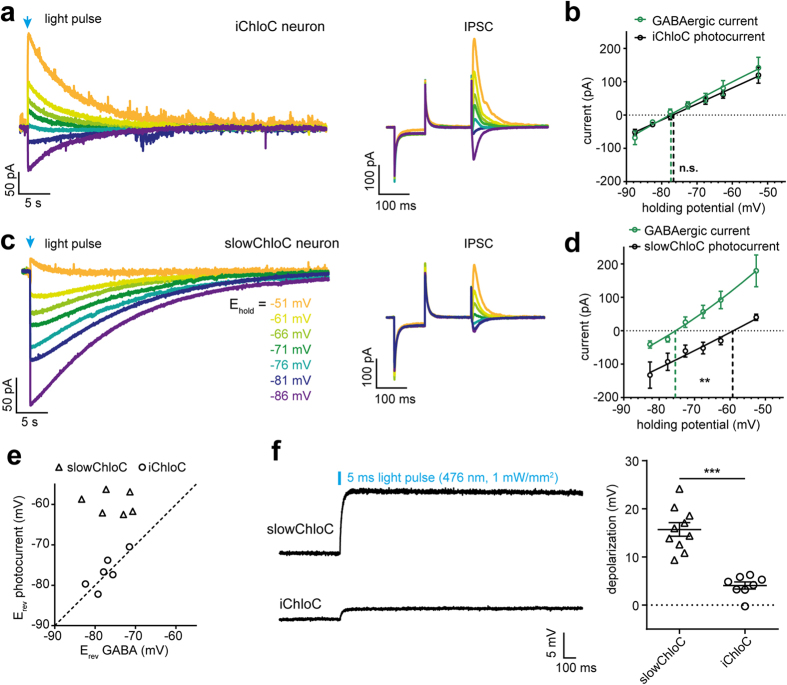
Photocurrent reversal potential vs. GABA receptor reversal potential. **(A)** Current traces of CA1 pyramidal cell expressing iChloC. Photocurrent reversal (left) is close to the reversal potential of GABA_A_ receptor mediated IPSCs (right), indicating pure Cl^−^ conductance. **(B)** I-E curves for iChloC photocurrents and IPSCs. **(C)** Current traces of CA1 pyramidal cell expressing slowChloC. The photocurrent (left) reverses more positive than GABA_A_ receptor mediated IPSCs (right), indicating mixed ionic conductance of slowChloC. **(D)** I-E curves for slowChloC photocurrents and IPSCs. **(E)** No systematic difference in GABA_A_ reversal between slowChloC and iChloC neurons. Between individual neurons, GABA_A_ reversal and iChloC reversal were highly correlated (r^2 ^= 0.67), indicating that the same ions are conducted. SlowChloC photocurrents were not correlated with GABA_A_ reversal potential (r^2 ^= 0.04). **(F)** Light stimulation experiments under current clamp conditions (I = 0) show significantly smaller depolarization from resting membrane potential in iChloC expressing neurons (n = 8) compared to slowChloC expressing neurons (n = 10).

The measured reversal potential of a chloride-conducting channel depends on the chloride concentration gradient and is therefore very sensitive to the composition of the recording solutions. To compare iChloC and slowChloC under near-physiological conditions we used artificial cerebrospinal fluid (ACSF) containing 2 mM Ca^2+^ and 1 mM Mg^2+^. Compared to our previous measurements in ACSF with a higher concentration of divalent ions^8^, the measured reversal potential of slowChloC was 8 mV more positive. To provide a biological calibration value, we stimulated inhibitory inputs to iChloC expressing neurons at various holding potentials (5–10 mV increments) while blocking glutamatergic transmission (10 μM NBQX, 10 μM CPPene). This allowed us to precisely determine the membrane voltage under which GABAergic currents reverse and to directly compare it to the reversal potential of iChloC (Fig. 4A). On average, IPSCs reversed at −77.3 ± 1.4 mV (Fig. 4B), close to the calculated Nernst potential for Cl^−^ (−75.9 mV). Light-induced iChloC currents reversed at −76.7 ± 1.6 mV, statistically not different from endogenous GABAergic inhibition (P = 0.6). SlowChloC photocurrents, in contrast, had a reversal potential of −59.7 ± 1.0 mV, significantly different from GABAergic inhibition in the same cells (−75.7 ± 1.8 mV, P = 0.0013), indicating a residual cation conductance (Fig. 4C,D). In contrast to the photocurrent reversal potentials, which significantly differed (P < 0.0001), the GABA_A_ reversal potential itself was not different between slowChloC and iChloC expressing neurons (Fig. 4E, P = 0.5). The difference in photocurrent reversal potential was also apparent in current clamp experiments, where slowChloC expressing neurons depolarized by 15.7 ± 1.3 mV from their resting membrane potential in response to a brief light pulse, while iChloC expressing neurons depolarized by only 4.4 ± 1.1 mV (Fig. 4F, P < 0.0001).

The recently discovered channelrhodopsin from *Chloromonas oogama*, CoChR, has been reported to produce very large photocurrents in neurons^19^. When we introduced the three pore mutations of iChloC into CoChR (E63Q, E70R, E81S), the reversal potential of photocurrents in HEK 293 cells was indeed very negative (E_rev _= −62 ± 3 mV; n = 8), indicating successful conversion into a chloride-conducting CoChR. However, photocurrents were smaller compared to iChloC (73 ± 15 pA; n = 8). In addition, expression of the chloride-conducting CoChR was not well tolerated by neurons, perhaps due to some leak current in the dark. We decided not to pursue CoChR-based variants further and focused on the complete characterization of iChloC effects in neurons.

**Figure 5:**
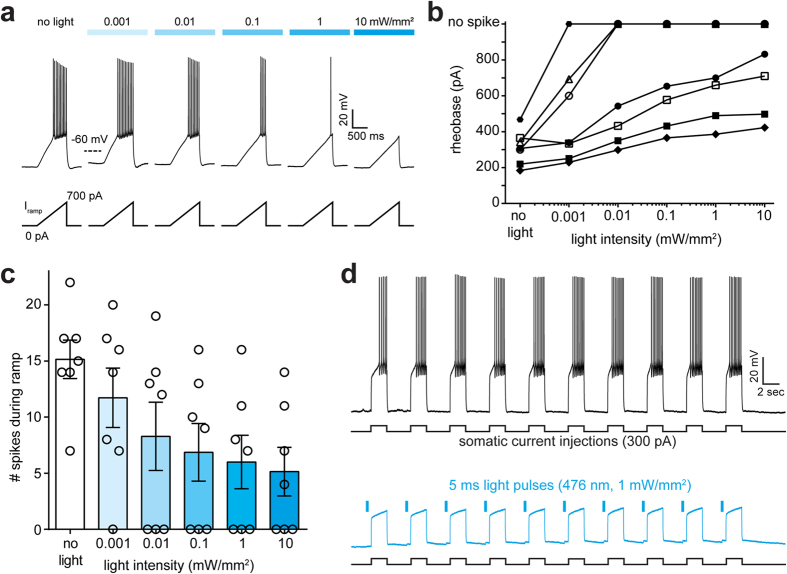
Spike suppression by iChloC. **(A)** Voltage traces in response to depolarizing current ramps (0–700 pA) injected into an iChloC-expressing CA1 pyramidal cell. The injected current at the time of the first spike was defined as the rheobase. Illumination with blue light activated iChloC. Increasing light intensities shifted the rheobase to higher values. **(B)** Individual neurons had different spike thresholds in the dark and showed different operational light sensitivity as a function of iChloC expression level. **(C)** Number of spikes during the ramp as a function of light intensity. At 0.1 mW/mm^2^, spike number was reduced to 45%, on average. **(D)** Suppression of depolarization-induced spiking by iChloC activation was highly reproducible. Due to the slow kinetics of iChloC, 5-ms light pulses were sufficient to block spiking for several seconds.

Next, we tested the efficacy and light sensitivity of spike inhibition by iChloC. Spikes were induced by depolarizing current ramps (from 0–100 to 0–1000 pA) injected into the somata of transfected CA1 pyramidal cells (Fig. 5A). In the dark, the rheobase varied between 184–468 pA, reflecting differences in input resistance and excitability of individual neurons (Fig. 5B). In all tested neurons, illumination shifted the rheobase to larger values, indicating a consistent inhibitory effect of iChloC activation. In strongly expressing neurons, spikes were completely blocked at light levels of 10 μW/mm^2^ and above (Fig. 5B). Averaging across the population of transfected neurons, 100 μW/mm^2^ reduced the spike output to 45% of control levels (Fig. 5C). Inhibitory performance of iChloC was reproducible and could be maintained for minutes by repetitive pulsed illumination (Fig. 5D).

**Figure 6:**
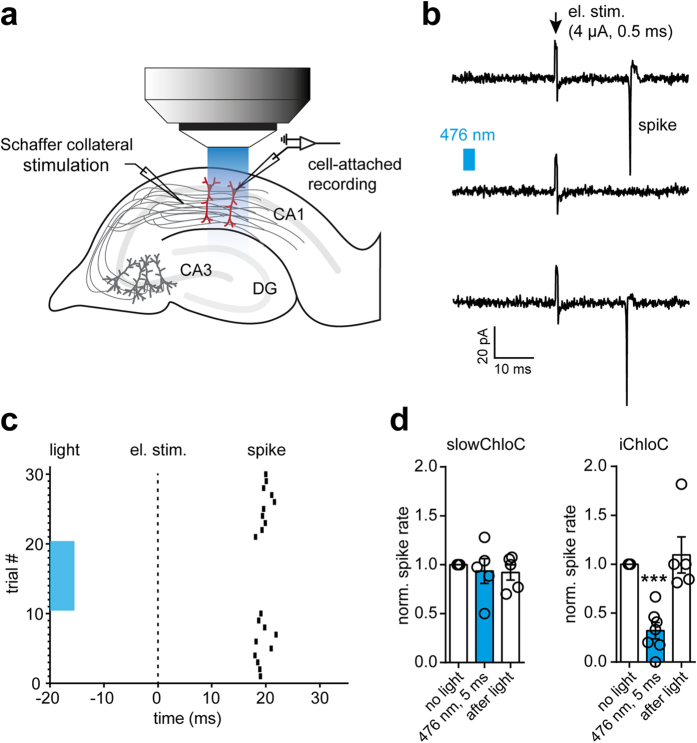
Suppression of synaptically evoked spikes by iChloC. (**A)** Schema of targeted cell-attached recordings from transfected CA1 pyramidal cells in an organotypic hippocampal slice culture. Drawing by J.S. Wiegert & T.G. Oertner. **(B)** Postsynaptic spikes were induced by electrical stimulation of afferents in *stratum radiatum* (4 μA, 0.5 ms). Upper trace: electrical stimulation, only. Middle trace: a blue light pulse (0.1 mW/mm^2^, 5 ms) delivered through the water immersion objective prevented spike initiation after synaptic stimulation. Lower trace: a spike was triggered again by electrical stimulation alone. **(C)** Raster plot of spike timing after synaptic stimulation of iChloC expressing neuron. Light pulse reliably blocked synaptically evoked spikes (trials #11–20). **(D)** Summary of synaptic stimulation experiments. While slowChloC activation had no significant effect on electrical spike induction (n = 5 slices cultures), iChloC activation blocked spiking in the majority of neurons (n = 7 slice cultures). Spike inhibition was fully reversible in all experiments.

In published whole-cell patch-clamp measurements, slowChloC blocked neuronal spiking with high efficiency despite its imperfect chloride selectivity and its depolarizing effect^8^. A limitation of whole-cell recordings is the disturbance of the intracellular milieu. Depending on the electrode solution used, the spike threshold might be different in patch-clamped neurons compared to unperturbed cells. To investigate optogenetic inhibition in unperturbed neurons, we performed cell-attached recordings from iChloC expressing CA1 pyramidal cells (Fig. 6A). Action potentials were induced at 0.1 Hz by electrical stimulation of afferents in *stratum radiatum*. Activation of iChloC by brief light pulses (5 ms) reliably suppressed synaptically induced action potentials (Fig. 6B). The inhibitory effect was fully reversible (Fig. 6C). On average, synaptically evoked action potentials were blocked with an efficiency of 68 ± 8% (P < 0.0001) in unperturbed CA1 neurons. In contrast, in CA1 neurons expressing slowChloC, no consistent inhibition or excitation was detected under these conditions (P = 0.75) (Fig. 6D).

**Figure 7:**
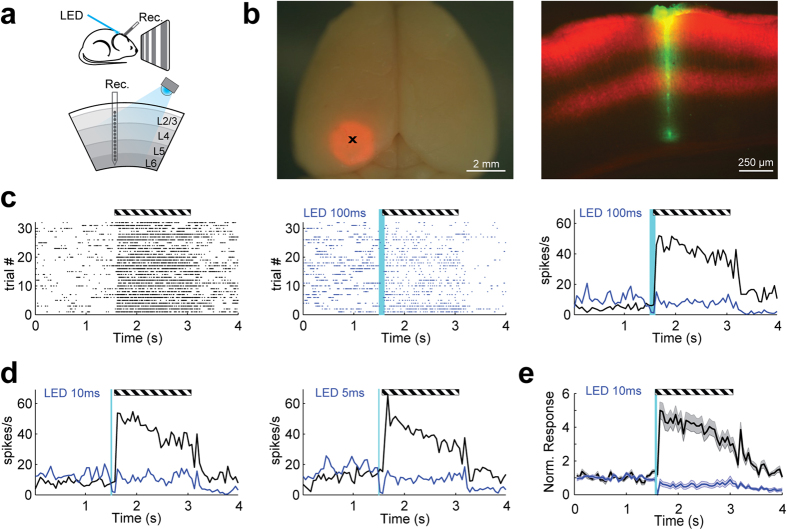
*In vivo* suppression of sensory evoked spikes by iChloC. **(A)** Schematic representation of the experimental setup. Drawing by R. Beltramo and M. Scanziani. **(B)** Left panel: Epifluorescence image of a mouse brain expressing iChloC (red) in V1. The black ‘x’ indicates the penetration site of the extracellular electrode that recorded the spikes shown in (**C**). Right panel: Epifluorescence image of coronal section through V1 of the brain shown on the left. Red: Viral expression of iChloC; Green: Recording site. **(C)** Extracellular recording from V1 in response to visual stimulation (full-field drifting gratings) under control conditions (black raster plot of multi-unit activity, left panel) and following 100 ms of iChloC photoactivation (blue raster plot, central panel). Control and photostimulation trials were interleaved. Right panel: Average peri-stimulus time histograms (PSTH, binning 50 ms) of control (black) and photostimulation (blue) trials. Striped horizontal bar indicates visual stimulus duration (1.5 s). Vertical cyan line: iChloC photoactivation (100 ms). **(D)** Same as in (C) for iChloC photostimulation of 10 ms and 5 ms durations. **(E)** Baseline normalized average PSTH (solid lines) and standard errors (shaded areas) of cortical responses to drifting gratings with (blue) and without (black) iChloC photoactivation (10 ms), from 4 tetrodes in 2 animals.

Finally we asked whether iChloC could be used to inhibit spiking activity of pyramidal neurons in the mammalian brain. We created a viral vector and infected the primary visual cortex in mice (Fig. 7B). Since iChloC-2A-tdimer2 was under control of a CaMKII-promoter, expression was largely restricted to pyramidal neurons (Fig. 7B). One month after virus injection, extracellular multi-unit activity was recorded with a multichannel linear electrode inserted in infragranular layers of the visual cortex of anaesthetized mice. Visual stimulation via presentation of drifting gratings for 1.5 s increased spiking activity approximately fourfold (Fig. 7C–E). A brief light flash delivered through an optical fiber to the surface of the brain (470 nm, 5–100 ms, 19 mW/mm^2^ at the fiber tip) immediately before, or coincident with the onset of the visual stimulus, completely blocked the increase in firing frequency (Fig. 7C–E). Compared to baseline, a 10 ms light flash reduced the average spiking frequency by 40.5 ± 15.5% during and 61.9 ± 5.8% after the visual stimulus (Fig. 7E). Thus, due to the slow off-kinetics and the high operational light sensitivity of iChloC, a brief light flash from an LED-coupled optical fiber pointing at the surface of the brain was sufficient to strongly suppress spiking activity for several seconds in visual cortex.

## Discussion

We developed a second-generation ChloC with improved Cl^−^ selectivity and demonstrate its function by silencing unperturbed neurons *in vitro* and *in vivo*. The first generation of ChloCs^8^,^9^ established that the ion preference of ChR2 could be radically changed from cations to anions, an unexpected discovery that constituted a major conceptual advance. However, the reversal potential of first-generation ChloCs was still slightly more positive than the reversal potential for Cl^−^ ions, indicating that cation conductivity was not completely eliminated. In consequence, light activation in neurons resulted in a small but significant depolarization from the resting membrane potential^8^. Here we show that first-generation slowChloC, in spite of its strong shunting effect in patch-clamped neurons^8^, is not able to reliably inhibit synaptically evoked action potentials in unperturbed neurons (Fig. 6D).

To faithfully inhibit free-running neurons, it was necessary to engineer out the remaining cation conductance to create a purely chloride-selective light-gated ion channel. This was achieved by removing two negatively charged residues from the aqueous pore of slowChloC - one in the access channel and one in the inner gate (Fig. 1). We estimate that the light intensity needed for half-maximal spike suppression by iChloC (0.1 mW/mm^2^) is two orders of magnitude lower compared to halorhodopsin or archaerhodopsin (10 mW/mm^2^, typically^6^). This difference in sensitivity translates to an increase in brain volume that can be inhibited from a single light source. For example, if 473 nm light at a power of 5 mW is delivered from a 0.22 NA fiber with a core diameter of 0.1 mm into mammalian brain tissue, the power density drops below 10 mW/mm^2^ at a depth of 0.45 mm and below 0.1 mW/mm^2^ at a depth of 2.2 mm^20^. Assuming spherical illuminated volumes, this translates to a 117-fold increase in inhibited volume under identical illumination conditions.

Light-driven pumps require constant illumination to maintain inhibition. In contrast, iChloC-mediated inhibition can be sustained by brief light pulses every 3–5 s, further reducing the effective light dose. The possibility of pulsed illumination is especially advantageous for experiments involving metal microelectrodes which are liable to produce light-induced voltage swings due to the Becquerel effect. Sustained inhibition in the dark means that circuit function and oscillations can be investigated electrophysiologically without the risk of light artifacts.

When designing optogenetic experiments, it has to be kept in mind that the magnitude of the effect will always be a function of the expression level of the optogenetic actuator. For iChloC, we observed an absolute spike block in strongly expressing neurons (Figs 5A and 6C) and a highly significant reduction in spike probability across the population (Figs 5B and 6D). In primary visual cortex, spike rates dropped well below resting levels in spite of visual stimulation, indicating that the output of a large majority of neurons was blocked for several seconds (Fig. 7). The slow off-kinetics of iChloC may be a limitation for experiments that require a very precise end of inhibition. For the future, it would be desirable to develop bistable versions of iChloC that can be switched back to the closed state with a brief light pulse of different color^21^. This would increase the temporal precision of neuronal silencing. In addition, to achieve even better brain penetration with light, red-shifted iChloCs are on the wish-list. In any case, further engineering efforts must preserve the high chloride selectivity, low dark current and good protein stability of iChloC to achieve high expression levels and successful inhibition.

## Methods

### HEK293 cell recordings

Human codon-adapted *ChR2-T159C* (*C2-TC*) gene was cloned into p-mCherry-N1 as described^22^. Additional point mutations were introduced using QuikChange (Agilent Technologies, Santa Clara, CA, USA). HEK293 cells were seeded onto coverslips at a concentration of 1.25 × 10^5^ cells ml^−1^ and supplemented with 1 μM *all trans*-retinal. Transient transfection was performed using Fugene HD (Roche, Mannheim, Germany) 36–48 h before measurement. Whole-cell patch clamp measurements were performed and signals were amplified and digitized using AxoPatch200B and DigiData1400. Light activation was achieved using a Polychrome V (TILL Photonics, Planegg, Germany) adjusted to 465 ± 7 nm. Activation light was coupled into the optical path of an Axiovert 100 microscope (Carl Zeiss, Jena, Germany) and regulated with a programmable shutter system (VS25 and VCM-D1, Vincent Associates, Rochester, NY, USA). The applied light intensity was 9.86 mW/mm^2^, measured after passing through all optics and coverslip with a calibrated optometer (P 9710, Gigahertz Optik, Türkenfeld, Germany). Measured light intensities are given for the illuminated field of the W Plan-Apochromat 40x/1.0 DIC objective (0.264 mm^2^). For all experiments external buffer solutions were exchanged by superfusion of at least 4 ml of the respective buffer into the measuring chamber (volume ~500 μl) while the fluid level was controlled by MPCU bath handler (Lorenz Messgerätebau, Katlenburg-Lindau, Germany). The buffer compositions for the pipette solution (10 mM Cl^−^) was as follows (in mM): 2MgCl_2_, 2CaCl_2_, 1KCl, 1CsCl, 10EGTA, 10HEPES, 110Na-Aspartate, whereas the bath solution (150mM Cl^−^) was composed of (in mM): 2MgCl_2_, 2CaCl_2_, 1KCl, 1CsCl, 10HEPES, 140NaCl. The pH of all buffers was adjusted with N-methyl-D-glucamine or citric acid to pH 7.20. The final osmolarity was adjusted to 320 mOsm for extracellular solutions and 290 mOsm for intracellular solutions. Patch clamp recordings were established under low chloride (10 mM) conditions to exclude chloride adulteration. Therefore 140 mM NaCl was replaced by 140 mM Na-Aspartate in the bath solution. After successful whole-cell formation the extracellular buffer was changed to 150 mM NaCl, resulting in a liquid junction potential (LJP) of 10.5 mV. Data was acquired using pClamp 10.4 (Clampex). The LJP correction was applied on-line. Patch pipettes where were pulled from borosilicate glass using a P97 micropipette puller (Sutter Instruments, Novato, CA, USA) followed by fire-polishing resulting in pipette resistances between 1.5 and 2.5 MΩ. All whole-cell recordings had a minimum membrane resistance of 500 MΩ (usually > 1 GΩ) whereas the access resistance was kept below 10 MΩ.

### Neuronal recordings in slice cultures

All ChR mutants were subcloned into identical neuron-specific expression vectors (pAAV backbone, human *CaMKII* promoter), followed by a 2A ribosomal skip sequence^23^ and the sequence for a red fluorescent protein (tdimer2, a gift from R.W. Tsien). We deposited the AAV-plasmid encoding iChloC-2A-tdimer2 on Addgene (Plasmid #66709). Organotypic slice cultures of rat hippocampus were prepared as described^24^ and transfected by single-cell electroporation after 14 days *in vitro* (DIV). Plasmids were each diluted to 20 ng/μl in K-gluconate–based solution consisting of (in mM): 135 K-gluconate, 4MgCl_2_, 4Na_2_-ATP, 0.4Na-GTP, 10Na_2_-phosphocreatine, 3 ascorbate, 0.02 Alexa Fluor 594, and 10 HEPES (pH 7.2). An Axoporator 800A (Molecular Devices) was used to deliver 50 hyperpolarizing pulses (−12 mV, 0.5 ms) at 50 Hz. At DIV 18–20, targeted patch-clamp recordings of transfected neurons were performed under visual guidance using a BX-51WI microscope (Olympus), a Multiclamp 700B amplifier (Axon Instruments), and Ephus software (HHMI Janelia Farm^25^). Patch pipettes with a tip resistance of 3–4 MΩ were filled with (in mM): 135 K-gluconate, 4MgCl_2_, 4Na_2_-ATP, 0.4Na-GTP, 10Na_2_-phosphocreatine, 3 ascorbate, 0.2 EGTA, and 10 HEPES (pH 7.2). Artificial cerebrospinal fluid (ACSF) consisted of (in mM): 135NaCl, 2.5KCl, 2CaCl_2_, 1MgCl_2_, 10Na-HEPES, 12.5D-glucose, 1.25NaH_2_PO_4_ (pH 7.4). In patch-clamp experiments without synaptic stimulation, synaptic currents were blocked with 10 μM CPPene, 10 μM NBQX, and 10 μM bicuculline (Tocris). For IPSC recordings, excitatory synaptic currents were blocked with 10 μM CPPene and 10 μM NBQX. Measurements were corrected for a liquid junction potential of −10.6 mV. An LED light engine (Spectra X, Lumencor) was used for epifluorescence excitation and delivery of light pulses (476 nm). Light intensity was measured in the object plane with a calibrated power meter (LaserCheck, Coherent) and divided by the illuminated field of the LUMPLFLN 60XW objective (0.134 mm^2^). Afferent Schaffer collateral axons were stimulated with a monopolar glass electrode connected to a stimulus isolator (IS4 stimulator, Scientific Devices).

### Virus injections and *in vivo* physiology

All animal experiments were carried out in accordance with the animal care and handling guidelines set forth by the University of California and all procedures to maintain and use mice were approved by the Institutional Animal Care and Use Committee at the University of California, San Diego. Mice were maintained on a reverse 12-h:12-h light:dark cycle with regular mouse chow and water *ad libitum*. An adeno-associated virus AAV2/9-CamKII-iChloC-2A-tdimer2 was produced by Ingke Braren and Kristin Bobsin at the HEXT core facility of the University Medical Center Hamburg-Eppendorf. Packaging and helper plasmids (p5E/9, pHelper) were provided by Julie C. Johnston, University of Pennsylvania, USA. The virus (titer 3.75*10^12^ gene copies/ml, 150 nl) was injected at a rate of 30 nl/min into the left primary visual cortex (2.5 mm lateral to the midline, 1 mm anterior to the lambda suture) of juvenile (1–2 months) mice at 500 μm depth, as previously described^26^.

*In vivo* recordings were performed 1 month after viral injections. The animals were anesthetized with isoflurane (1%–2.5%) and chlorprothixene (5 mg/kg), their temperature was maintained at 37 °C with a heating pad (FHC) and the eyes were covered by a thin layer of silicone oil to prevent drying. A head-plate was mounted on the hemisphere contralateral to the injection site, using dental cement. The skull covering V1 was thinned, the extension of the viral infection was verified by transcranial epifluorescence, and a small craniotomy (250 μm diameter) for the probe insertion was made over V1. The dura was left intact and kept moist with artificial cerebrospinal fluid (ACSF; 140 mM NaCl, 5 mM KCl, 10 mM D-glucose, 10 mM HEPES, 2 mM CaCl_2_, 2 mM MgSO_4_, pH 7.4). Isoflurane was adjusted to 0.5% following completion of the surgery, and a NeuroNexus 16-channel linear probe (A1 × 16-5 mm-25–177-A16) was inserted in the cortex at a depth of 700–900 μm from the pial surface. Electric signals were amplified X 2000 and band-pass filtered (0.3 Hz–5 kHz) using an AM System 3500 amplifier. The recordings were acquired at 32 kHz using a NIDAQ PCIe-6239 board controlled by custom-written MATLAB software (MathWorks). At the end of the recording session, the electrode was gently extracted from the brain, dipped into DiO cell-labeling solution (Life Technologies) and reinserted into the cortex at the same coordinates used for the recordings. Animals were then transcardially perfused with PBS (pH 7.4) followed by 4% paraformaldehyde in PBS. Brains were further fixed overnight in 4% paraformaldehyde and sectioned to obtain 100 μm coronal slices. Slices were mounted in Vectashield Mounting Medium and images acquired on an Olympus MVX10 Macroview.

### Visual stimulation and optogenetic silencing *in vivo*

Visual stimuli were generated by a custom-written Matlab software provided by M. Caudill, and displayed on a gamma-corrected LCD monitor (Dell, 48 × 30 cm, 60-Hz refresh rate, mean luminance 50 cd m^−2^) positioned 15 cm from the eye contralateral to the craniotomy. Full-field sinusoidal drifting gratings (50% and 100% contrast, spatial frequency of 0.04 and 0.08 cycles per degree, temporal frequency of 1 and 4 Hz) were displayed at 2 orthogonal orientations (0° and 90°) for 1.5 s, preceded and followed by the presentation of a grey screen of mean luminance for 1.5 s and 5 s, respectively.

To photostimulate iChloC, a blue LED (470 nm, Thorlabs) was coupled to a fiber optic (1.0 mm diameter, Thorlabs) placed over V1. The light was presented at 15 mW (corresponds to 19 mW/mm^2^ at the fiber tip) in pulses of 5 ms, 10 ms, 50 ms and 100 ms duration. Trials with visual stimulus only were interleaved with trials with visual stimulus and iChloC photoactivation.

### Data analysis

Data were analyzed in Matlab, GraphPad Prism, Clampfit 10.4 or Origin 9. All data are given as mean ± standard error of the mean. The following statistical tests were used: Student’s t-test (unpaired, two-tailed) for data shown in Fig. 4B,D,E,F. One-way ANOVA followed by Dunett’s multiple comparison test for data shown in Fig. 6D. Significance levels are indicated as follows: **(P ≤ 0.01), ***(P ≤ 0.001), n.s. (not significant). Exact P values are given in the main text. P values smaller than 0.0001 are given as “P < 0.0001”. *In vivo* data analysis was performed using custom software written in Matlab and spikes were isolated as described before^26^, using software provided by D. N. Hill, S. B. Mehta, and D. Kleinfeld^27^. Action potentials were defined as events exceeding 4 SD of the noise, and the detected spikes from all stimulus conditions were pooled together to generate raster plots and average peri-stimulus time histograms (PSTH, binning 50 ms) of the multi-unit activity (MU).

## Additional Information

**How to cite this article**: Wietek, J. *et al.* An improved chloride-conducting channelrhodopsin for light-induced inhibition of neuronal activity in vivo. *Sci. Rep.* **5**, 14807; doi: 10.1038/srep14807 (2015).

## Figures and Tables

**Table 1:** Neuronal membrane parameters.

	iChloC (n = 9)	S.D.	wt (n = 9)	S.D.	*P*
AP threshold (mV)	−38.77	5.37	−43.42	7.08	*0.16*
AP peak voltage (mV)	36.19	3.70	35.81	2.22	*0.81*
AP amplitude (mV)	117.52	4.04	111.06	9.94	*0.11*
n AP’s	11.22	4.47	14.44	1.83	*0.08*
E_rest_ (mV)	−81.33	3.39	−75.25	10.52	*0.14*
R_M_ (Mohm)	181.01	39.75	144.90	54.81	*0.20*

Electrical parameters of untransfected pyramidal CA1 neurons and neurons expressing iChloC together with tdimer2. Action potentials (APs) were evoked by a square current pulse (500 ms, 500 pA) in current clamp (IC) mode. Threshold, peak voltage and amplitude were calculated for the first AP. Membrane resistance (R_M_) was measured in voltage clamp mode in response to a square voltage pulse (−5 mV, 100 ms). E_rest _= resting membrane potential, S.D. = standard deviation. Right column indicates P values from unpaired t-test for each parameter. No significant differences were detected. All measurements were liquid junction potential corrected.
